# Sharp bounds for a special quasi-arithmetic mean in terms of arithmetic and geometric means with two parameters

**DOI:** 10.1186/s13660-017-1550-5

**Published:** 2017-11-02

**Authors:** Wei-Mao Qian, Yu-Ming Chu

**Affiliations:** 1 School of Distance Education, Huzhou Broadcast and TV University, Huzhou, 313000 China; 2 Department of Mathematics, Huzhou University, Huzhou, 313000 China

**Keywords:** 26E60, 33E05, quasi-arithmetic mean, complete elliptic integral, Gaussian hypergeometric function, arithmetic mean, geometric mean

## Abstract

In the article, we present the best possible parameters $\lambda=\lambda (p)$ and $\mu=\mu(p)$ on the interval $[0, 1/2]$ such that the double inequality

$$\begin{aligned}& G^{p}\bigl[\lambda a+(1-\lambda)b, \lambda b+(1-\lambda)a \bigr]A^{1-p}(a,b) \\& \quad< E(a,b) < G^{p}\bigl[\mu a+(1-\mu)b, \mu b+(1-\mu)a \bigr]A^{1-p}(a,b) \end{aligned}$$

holds for any $p\in[1, \infty)$ and all $a, b>0$ with $a\neq b$, where $A(a, b)=(a+b)/2$, $G(a,b)=\sqrt{ab}$ and $E(a,b)=[2\int_{0}^{\pi /2}\sqrt{a\cos^{2}\theta+b\sin^{2}\theta}\,d\theta/\pi]^{2}$ are the arithmetic, geometric and special quasi-arithmetic means of *a* and *b*, respectively.

## Introduction

Let $r\in(0,1)$. Then the Legendre complete elliptic integrals $\mathcal {K}(r)$ and $\mathcal{E}(r)$ [1, 2] of the first and second kinds are defined as

$$ \mathcal{K}(r)= \int_{0}^{\pi/2}\frac{dt}{\sqrt{1-r^{2}\sin^{2}(t)}}, \qquad\mathcal{E}(r)= \int_{0}^{\pi/2}\sqrt{1-r^{2} \sin^{2}(t)}\,dt, $$

respectively. It is well known that the function $r\rightarrow\mathcal {K}(r)$ is strictly increasing from $(0, 1)$ onto $(\pi/2, \infty)$ and the function $r\rightarrow\mathcal{E}(r)$ is strictly decreasing from $(0, 1)$ onto $(1, \pi/2)$, and they satisfy the formulas (see [3, Appendix E, pp. 474,475])

$$ \begin{gathered} \frac{d{\mathcal{K}(r)}}{dr}=\frac{{\mathcal{E}(r)}-{r'}^{2}{\mathcal {K}}(r)}{r{r'}^{2}},\qquad \frac{d{\mathcal{E}(r)}}{dr}=\frac{{\mathcal{E}(r)}-{\mathcal{K}(r)}}{r}, \\ \mathcal{K} \biggl(\frac{2\sqrt{r}}{1+r} \biggr)=(1+r)\mathcal{K}(r),\qquad \mathcal{E} \biggl(\frac{2\sqrt{r}}{1+r} \biggr)=\frac{2\mathcal {E}(r)-{r'}^{2}\mathcal{K}}{1+r}, \end{gathered} $$

where $r'=\sqrt{1-r^{2}}$.

The complete elliptic integrals $\mathcal{K}(r)$ and $\mathcal{E}(r)$ are the particular cases of the Gaussian hypergeometric function [4–10]

$$ F(a,b;c;x)=\sum_{n=0}^{\infty}\frac{(a)_{n}(b)_{n}}{(c)_{n}} \frac {x^{n}}{n!}\quad (-1< x< 1), $$

where $(a)_{0}=1$ for $a\neq0$, $(a)_{n}=a(a+1)(a+2)\cdots (a+n-1)=\Gamma(a+n)/\Gamma(a)$ is the shifted factorial function and $\Gamma(x)=\int_{0}^{\infty }t^{x-1}e^{-t}\,dt$ ($x>0$) is the gamma function [11–18]. Indeed,

$$ \begin{gathered} \mathcal{K}(r)=\frac{\pi}{2}F \biggl( \frac{1}{2},\frac{1}{2};1;r^{2} \biggr) = \frac{\pi}{2}\sum_{n=0}^{\infty} \frac{ (\frac{1}{2} )_{n}^{2}}{(n!)^{2}}r^{2n}, \\ \mathcal{E}(r)=\frac{\pi}{2}F \biggl(-\frac{1}{2}, \frac{1}{2};1;r^{2} \biggr) =\frac{\pi}{2}\sum _{n=0}^{\infty}\frac{ (-\frac{1}{2} )_{n} (\frac{1}{2} )_{n}}{(n!)^{2}}r^{2n}. \end{gathered} $$

Recently, the bounds for the complete elliptic integrals have attracted the attention of many researchers. In particular, many remarkable inequalities and properties for $\mathcal{K}(r)$, $\mathcal{E}(r)$ and $F(a,b;c;x)$ can be found in the literature [19–52].

In 1998, a class of quasi-arithmetic mean was introduced by Toader [53] which is defined by

$$ M_{p,n}(a,b)=p^{-1} \biggl(\frac{1}{\pi} \int_{0}^{\pi}p\bigl(r_{n}(\theta )\,d \theta\bigr) \biggr)=p^{-1} \biggl(\frac{2}{\pi} \int_{0}^{\pi/2}p\bigl(r_{n}(\theta )\,d \theta\bigr) \biggr), $$

where $r_{n}(\theta)=(a^{n}\cos^{2}\theta+b^{n}\sin^{2}\theta)^{1/n}$ for $n\neq0$, $r_{0}(\theta)=a^{\cos^{2}\theta}b^{\sin^{2}\theta}$, and *p* is a strictly monotonic function. It is well known that many important means are the special cases of the quasi-arithmetic mean. For example,

$$\begin{aligned} M_{1/x,2}(a,b)= \frac{\pi}{2 \int_{0}^{\pi /2}{\frac{d\theta}{\sqrt{a^{2}\cos^{2}\theta+b^{2}\sin^{2}\theta}}}} = \textstyle\begin{cases} {\pi a} / [2{\mathcal{K}} (\sqrt{1-(b/a)^{2}} ) ],&a\geq b,\\ {\pi b} / [2{\mathcal{K}} (\sqrt{1-(a/b)^{2}} ) ],&a< b, \end{cases}\displaystyle \end{aligned}$$

is the arithmetic-geometric mean of Gauss [54–60],

$$ M_{x,2}(a,b))=\frac{2}{\pi} \int_{0}^{\pi/2}\sqrt{a^{2}\cos^{2} \theta +b^{2}\sin^{2}\theta}\,d\theta = \textstyle\begin{cases}2a{\mathcal{E}} (\sqrt{1-(b/a)^{2}} )/\pi,&a\geq b,\\ 2b{\mathcal{E}} (\sqrt{1-(a/b)^{2}} )/\pi,&a< b, \end{cases} $$

is the Toader mean [61–70], and

$$ M_{x,0}(a,b))=\frac{2}{\pi} \int_{0}^{\pi/2}a^{\cos^{2}\theta}b^{\sin ^{2}\theta}\,d \theta $$

is the Toader-Qi mean [71–74].

Let $p=\sqrt{x}$ and $n=1$. Then $M_{p,n}(a,b)$ reduces to a special quasi-arithmetic mean

1.1 $$ E(a,b)=M_{\sqrt{x},1}(a,b))= \textstyle\begin{cases}4a [{\mathcal{E}} (\sqrt{1-b/a} ) ]^{2}/\pi ^{2},&a\geq b,\\ 4b [{\mathcal{E}} (\sqrt{1-a/b} ) ]^{2}/\pi^{2},&a< b. \end{cases} $$

Let

$$ \begin{gathered} A(a,b)=\frac{a+b}{2}, \qquad G(a,b)=\sqrt{ab}, \\ M_{p}(a,b)= \biggl(\frac{a^{p}+b^{p}}{2} \biggr)^{1/p} (p\neq0), \qquad M_{0}(a,b)=\sqrt{ab}, \end{gathered} $$

be the arithmetic, geometric and *p*th power means of *a* and *b*, respectively. Then it is well known that the inequality

1.2 $$ G(a,b)=M_{0}(a,b)< A(a,b)=M_{1}(a,b) $$

holds for all $a, b>0$ with $a\neq b$, and the double inequality

1.3 $$ \frac{\pi}{2}M_{3/2}\bigl(1, r^{\prime}\bigr)< \mathcal{E}(r)< \frac{\pi }{2}M_{2}\bigl(1, r^{\prime}\bigr) $$

holds for all $r\in(0, 1)$ (see [75, 19.9.4]).

From (1.1)-(1.3) we clearly see that

$$ G(a,b)< E(a,b)< A(a,b) $$

for all $a, b>0$ with $a\neq b$.

Let $p\in[1, \infty)$ and

$$ f(x; p; a, b)=G^{p}\bigl[xa+(1-x)b, xb+(1-x)a\bigr]A^{1-p}(a,b). $$

Then it is not difficult to verify that the function $x\rightarrow f(x; p; a, b)$ is strictly increasing on $[0, 1/2]$ for fixed $p\in[1, \infty)$ and $a, b>0$ with $a\neq b$. Note that

1.4 $$ \begin{aligned}[b] f(0; p; a, b)&=G^{p}(a,b)A^{1-p}(a,b) \leq G(a,b) \\ &< E(a,b)< A(a,b)=f(1/2; p; a, b) \end{aligned} $$

for all $p\in[1, \infty)$ and $a, b>0$ with $a\neq b$.

Motivated by inequalities (1.4) and the monotonicity of the function $x\rightarrow f(x; p; a, b)$ on the interval $[0, 1/2]$, in the article, we shall find the best possible parameters $\lambda=\lambda(p), \mu=\mu(p)$ on the interval $[0, 1/2]$ such that the double inequality

$$ \begin{aligned} &G^{p}\bigl[\lambda a+(1-\lambda)b, \lambda b+(1-\lambda)a\bigr]A^{1-p}(a,b) \\ &\quad< E(a,b)< G^{p}\bigl[\mu a+(1-\mu)b, \mu b+(1-\mu)a \bigr]A^{1-p}(a,b) \end{aligned} $$

holds for any $p\in[1, \infty)$ and all $a, b>0$ with $a\neq b$.

## Lemmas

### Lemma 2.1

(see [3, Theorem 1.25])

*Let* $-\infty< a< b<+\infty$, $f, g: [a, b]\rightarrow\mathbb{R}$ *be continuous on* $[a, b]$ *and differentiable on* $(a,b)$, *and* $g^{\prime}(x)\neq0$ *on* $(a, b)$. *If* $f^{\prime}(x)/g^{\prime}(x)$ *is increasing* (*decreasing*) *on* $(a,b)$, *then so are the functions*

$$ \frac{f(x)-f(a)}{g(x)-g(a)}, \qquad\frac{f(x)-f(b)}{g(x)-g(b)}. $$

*If* $f^{\prime}(x)/g^{\prime}(x)$ *is strictly monotone*, *then the monotonicity in the conclusion is also strict*.

### Lemma 2.2

*The inequality*

$$ \frac{1}{4p}+ \biggl(\frac{2\sqrt{2}}{\pi} \biggr)^{4/p}< 1 $$

*holds for all* $p\in[1, \infty)$.

### Proof

Let

2.1 $$ f(p)=\frac{1}{4p}+ \biggl(\frac{2\sqrt{2}}{\pi} \biggr)^{4/p}. $$

Then simple computations lead to

2.2 $$\begin{aligned}& \lim_{p\rightarrow\infty}f(p)=1, \end{aligned}$$

2.3 $$\begin{aligned}& \begin{aligned}[b] f^{\prime}(p)&=\frac{4}{p^{2}}\log \biggl(\frac{\sqrt{2}\pi}{4} \biggr) \biggl[ \biggl(\frac{2\sqrt{2}}{\pi} \biggr)^{4/p}-\frac{1}{16\log (\frac{\sqrt{2}\pi}{4} )} \biggr] \\ &\geq\frac{4}{p^{2}}\log \biggl(\frac{\sqrt{2}\pi}{4} \biggr) \biggl[ \biggl( \frac{2\sqrt{2}}{\pi} \biggr)^{4}-\frac{1}{16\log (\frac {\sqrt{2}\pi}{4} )} \biggr] \\ &=\frac{1024\log (\frac{\sqrt{2}\pi}{4} )-\pi^{4}}{4\pi^{4}p^{2}}>0 \end{aligned} \end{aligned}$$

for $p\in[1, \infty)$.

Therefore, Lemma 2.2 follows easily from (2.1)-(2.3). □

### Lemma 2.3

*The following statements are true*:

1. *The function* $r\mapsto[\mathcal{E}(r)-(1-r^{2})\mathcal {K}(r)]/r^{2}$ *is strictly increasing from* $(0, 1)$ *onto* $(\pi/4, 1)$.
2. *The function* $r\mapsto[\mathcal{K}(r)-\mathcal {E}(r)]/r^{2}$ *is strictly increasing from* $(0, 1)$ *onto* $(\pi/4, \infty)$.
3. *The function* $r\mapsto[\mathcal{E}(r)+(1-r^{2})\mathcal {K}(r)]/(1-r^{2})$ *is strictly increasing from* $(0, 1)$ *onto* $(\pi, \infty)$.
4. *The function* $r\mapsto[2\mathcal{E}(r)-(1-r^{2})\mathcal {K}(r)]/(1+r^{2})$ *is strictly decreasing from* $(0, 1)$ *onto* $(1, \pi/2)$.
5. *The function* $r\mapsto r^{2}[2\mathcal {E}(r)-(1-r^{2})\mathcal{K}(r)]/ [(1+r^{2})^{2}(\mathcal {K}(r)-\mathcal{E}(r)) ]$ *is strictly decreasing from* $(0, 1)$ *onto* $(0, 2)$.

### Proof

Parts (1) and (2) can be found in the literature [3, Theorem 3.21(1) and Exercise 3.43(11)].

For part (3), let $f_{1}(r)=[\mathcal{E}(r)+(1-r^{2})\mathcal {K}(r)]/(1-r^{2})$. Then simple computations lead to

2.4 $$\begin{aligned}& f_{1}\bigl(0^{+}\bigr)=\pi, \qquad f_{1} \bigl(1^{-}\bigr)=\infty, \end{aligned}$$

2.5 $$\begin{aligned}& f^{\prime}_{1}(r)=\frac{r}{(1-r^{2})^{2}} \biggl[\frac{2}{r^{2}} \bigl(\mathcal {E}(r)-\bigl(1-r^{2}\bigr)\mathcal{K}(r)\bigr)+ \bigl(1-r^{2}\bigr)\mathcal{K}(r) \biggr]. \end{aligned}$$

It follows from part (1) and (2.5) that

2.6 $$ f^{\prime}_{1}(r)>0 $$

for all $r\in(0, 1)$. Therefore, part (3) follows from (2.4) and (2.6).

For part (4), let $f_{2}(r)=[2\mathcal{E}(r)-(1-r^{2})\mathcal {K}(r)]/(1+r^{2})$, then one has

2.7 $$\begin{aligned}& f_{2}\bigl(0^{+}\bigr)=\frac{\pi}{2}, \qquad f_{1}\bigl(1^{-}\bigr)=1, \end{aligned}$$

2.8 $$\begin{aligned}& f^{\prime}_{2}(r)=\frac{r}{(1+r^{2})^{2}} \biggl[ \bigl(1-r^{2}\bigr)\frac{\mathcal {E}(r)-(1-r^{2})\mathcal{K}(r)}{r^{2}}-2\mathcal{E}(r) \biggr]. \end{aligned}$$

From part (1) and (2.8) we clearly see that

2.9 $$ f^{\prime}_{2}(r)< -\frac{r}{(1+r^{2})}< 0 $$

for all $r\in(0, 1)$. Therefore, part (4) follows from (2.7) and (2.9).

For part (5), let $f_{3}(r)=r^{2}[2\mathcal{E}(r)-(1-r^{2})\mathcal {K}(r)]/ [(1+r^{2})^{2}(\mathcal{K}(r)-\mathcal{E}(r)) ]$, then $f_{3}(r)$ can be rewritten as

2.10 $$ f_{3}(r)=\frac{2\mathcal{E}(r)-(1-r^{2})\mathcal{K}(r)}{1+r^{2}} \times\frac{1}{\frac{\mathcal{K}(r)-\mathcal{E}(r)}{r^{2}}}\times \frac {1}{1+r^{2}}. $$

Therefore, part (5) follows easily from parts (2) and (4) together with (2.10). □

### Lemma 2.4

*The function*

$$ g(r)=\frac{r^{2}\mathcal{K}(r)}{(1+r^{2})[\mathcal{K}(r)-\mathcal{E}(r)]} $$

*is strictly decreasing from* $(0, 1)$ *onto* $(1/2, 2)$.

### Proof

Let $g_{1}(r)=r^{2}\mathcal{K}(r)$ and $g_{2}(r)=(1+r^{2})[\mathcal {K}(r)-\mathcal{E}(r)]$. Then we clearly see that

2.11 $$\begin{aligned}& g_{1}\bigl(0^{+}\bigr)=g_{2} \bigl(0^{+}\bigr)=0, \qquad g(r)=\frac{g_{1}(r)}{g_{2}(r)}, \end{aligned}$$

2.12 $$\begin{aligned}& g\bigl(1^{-}\bigr)=\frac{1}{2}, \end{aligned}$$

2.13 $$\begin{aligned}& \frac{g^{\prime}_{1}(r)}{g^{\prime}_{2}(r)}=\frac{1}{2-\frac{3\mathcal {E}(r)}{\frac{\mathcal{E}(r)+(1-r^{2})\mathcal{K}(r)}{1-r^{2}}}}. \end{aligned}$$

From Lemma 2.3(3), (2.11) and (2.13) we know that

2.14 $$ g\bigl(0^{+}\bigr)=\lim_{r\rightarrow0^{+}}\frac{g^{\prime}_{1}(r)}{g^{\prime}_{2}(r)}=2 $$

and the function $g^{\prime}_{1}(r)/g^{\prime}_{2}(r)$ is strictly decreasing on $(0, 1)$.

Therefore, Lemma 2.4 follows easily from Lemma 2.1, (2.11), (2.12) and (2.14) together with the monotonicity of the function $g^{\prime}_{1}(r)/g^{\prime}_{2}(r)$. □

### Lemma 2.5

*Let* $u\in[0, 1]$, $r\in(0, 1)$, $p\in[1, \infty)$ *and*

2.15 $$ h(u, p; r)=\frac{1}{2}p\log \biggl[1-\frac{4ur^{2}}{(1+r^{2})^{2}} \biggr] -\log \biggl[\frac{4 (2\mathcal{E}(r)-(1-r^{2})\mathcal{K}(r) )^{2}}{\pi^{2}(1+r^{2})} \biggr]. $$

*Then one has*

1. $h(u, p; r)>0$ *for all* $r\in(0, 1)$ *if and only if* $u\leq1/4p$;
2. $h(u, p; r)<0$ *for all* $r\in(0, 1)$ *if and only if* $u\geq 1-(2\sqrt{2}/\pi)^{4/p}$.

### Proof

It follows from (2.15) that

2.16 $$\begin{aligned}& h\bigl(u, p; 0^{+}\bigr)=0, \end{aligned}$$

2.17 $$\begin{aligned}& h\bigl(u, p; 1^{-}\bigr)=\frac{p}{2}\log(1-u)+\log \biggl( \frac{\pi^{2}}{8} \biggr), \end{aligned}$$

2.18 $$\begin{aligned}& \begin{aligned}[b] \frac{\partial h(u, p; r)}{\partial r}&=\frac{2(1-r^{2})[\mathcal {K}(r)-\mathcal{E}(r)]}{ r(1+r^{2})[2\mathcal{E}(r)-(1-r^{2})\mathcal{K}(r)]} - \frac{4pur(1-r^{2})}{(1+r^{2}) [(1+r^{2})^{2}-4ur^{2} ]} \\ &=\frac{2(1-r^{2}) [2(\mathcal{K}(r)-\mathcal {E}(r))+p(2\mathcal{E}(r)-(1-r^{2})\mathcal{K}(r)) ]}{(1+r^{2}) [(1+r^{2})^{2}-4ur^{2} ][2\mathcal {E}(r)-(1-r^{2})\mathcal{K}(r)]}\bigl[h_{1}(p; r)-2u\bigr], \end{aligned} \end{aligned}$$

where

2.19 $$ \begin{aligned}[b] h_{1}(p; r)&=\frac{(1+r^{2})^{2}[\mathcal{K}(r)-\mathcal{E}(r)]}{r^{2} [2(\mathcal{K}(r)-\mathcal{E}(r))+p(2\mathcal {E}(r)-(1-r^{2})\mathcal{K}(r)) ]} \\ &=\frac{1}{g(r)+(p-1)f_{3}(r)}, \end{aligned} $$

where $f_{3}(r)$ and $g(r)$ are defined by (2.10) and Lemma 2.4, respectively.

From Lemma 2.3(5) and Lemma 2.4 together with (2.19) we clearly see that the function $r\rightarrow h_{1}(p; r)$ is strictly increasing on $(0, 1)$ and

2.20 $$\begin{aligned}& h_{1}\bigl(p; 0^{+}\bigr)=\frac{1}{2p}, \end{aligned}$$

2.21 $$\begin{aligned}& h_{1}\bigl(p; 1^{-}\bigr)=2. \end{aligned}$$

From Lemma 2.2 we know that $1-(2\sqrt{2}/\pi)^{4/p}>1/(4p)$. Therefore, we only need to divide the proof into three cases as follows.

*Case 1* $u\leq1/(4p)$. Then Lemma 2.3(4), (2.18), (2.20) and the monotonicity of the function $r\rightarrow h_{1}(p; r)$ on the interval $(0, 1)$ lead to the conclusion that the function $r\rightarrow h(u, p; r)$ is strictly increasing on $(0, 1)$. Therefore, $h(u, p; r)>0$ for all $r\in(0, 1)$ follows from (2.16) and the monotonicity of the function $r\rightarrow h(u, p; r)$.

*Case 2* $u\geq1-(2\sqrt{2}/\pi)^{4/p}$. Then from Lemma 2.2, Lemma 2.3(5), (2.17), (2.18), (2.20), (2.21) and the monotonicity of the function $r\rightarrow h_{1}(p; r)$ on the interval $(0, 1)$ we clearly see that there exists $r_{0}\in(0, 1)$ such that the function $r\rightarrow h(u, p; r)$ is strictly decreasing on $(0, r_{0})$ and strictly increasing on $(r_{0}, 1)$, and

2.22 $$ h\bigl(u, p; 1^{-}\bigr)\leq0. $$

Therefore, $h(u, p; r)<0$ for all $r\in(0, 1)$ follows from (2.16) and (2.22) together with the piecewise monotonicity of the function $r\rightarrow h(u, p; r)$ on the interval $(0, 1)$.

*Case 3* $1/(4p)< u<1-(2\sqrt{2}/\pi)^{4/p}$. Then (2.17) leads to

2.23 $$ h\bigl(u, p; 1^{-}\bigr)>0. $$

It follows from Lemma 2.3(5), (2.18), (2.20), (2.21) and the monotonicity of the function $r\rightarrow h_{1}(p; r)$ on the interval $(0, 1)$ that there exists $r^{\ast}\in(0, 1)$ such that the function $r\rightarrow h(u, p; r)$ is strictly decreasing on $(0, r^{\ast})$ and strictly increasing on $(r^{\ast}, 1)$. Therefore, there exists $\lambda \in(0, 1)$ such that $h(u, p; r)<0$ for $r\in(0, \lambda)$ and $h(u, p; r)>0$ for $r\in(\lambda, 1)$. □

## Main result

### Theorem 3.1

*Let* $\lambda, \mu\in[0, 1/2]$. *Then the double inequality*

$$ \begin{gathered} G^{p}\bigl[\lambda a+(1-\lambda)b, \lambda b+(1-\lambda)a\bigr]A^{1-p}(a,b) \\ \quad< E(a,b)< G^{p}\bigl[\mu a+(1-\mu)b, \mu b+(1-\mu)a \bigr]A^{1-p}(a,b) \end{gathered} $$

*holds for any* $p\in[1, \infty)$ *and all* $a, b>0$ *with* $a\neq b$ *if and only if* $\lambda\leq1/2-\sqrt{1-(2\sqrt{2}/\pi)^{4/p}}/2$ *and* $\mu\geq1/2-\sqrt{p}/(4p)$.

### Proof

Let $t\in[0, 1/2]$, since $G^{p}[ta+(1-t)b, tb+(1-t)a]A^{1-p}(a,b)$ and $E(a,b)$ are symmetric and homogeneous of degree one, without loss of generality, we assume that $a>b>0$. Let $r\in(0, 1)$ and $b/a=(1-r)^{2}/(1+r)^{2}$. Then (1.1) leads to

3.1 $$ \begin{gathered} E(a,b)=\frac{4(1+r)^{2}}{\pi^{2}(1+r^{2})}A(a,b){\mathcal{E}}^{2} \biggl( \frac{2\sqrt{r}}{1+r} \biggr) =\frac{4}{\pi^{2}}A(a,b)\frac{ [2\mathcal{E}(r)-(1-r^{2})\mathcal {K}(r) ]^{2}}{1+r^{2}}, \\ \log \bigl[G^{p}\bigl(ta+(1-t)b, tb+(1-t)a \bigr)A^{1-p}(a,b) \bigr]-\log E(a,b) \\ \quad=\log \biggl[\frac{G^{p}(ta+(1-t)b, tb+(1-t)a)A^{1-p}(a,b)}{A(a,b)} \biggr]-\log \biggl[\frac {E(a,b)}{A(a,b)} \biggr] \\ \quad=\frac{1}{2}p\log \biggl[1-\frac {4(1-2t)^{2}r^{2}}{(1+r^{2})^{2}} \biggr] -\log \biggl[ \frac{4 (2\mathcal{E}(r)-(1-r^{2})\mathcal{K}(r) )^{2}}{\pi^{2}(1+r^{2})} \biggr]. \end{gathered} $$

Therefore, Theorem 3.1 follows easily from Lemma 2.5 and (3.1). □

Let $p=1, 2$, then Theorem 3.1 leads to Corollary 3.2 immediately.

### Corollary 3.2

*Let* $\lambda_{1}, \mu_{1}, \lambda_{2}, \mu_{2}\in[0, 1/2]$. *Then the double inequalities*

$$ \begin{gathered} H\bigl[\lambda_{1}a+(1- \lambda_{1})b, \lambda_{1}b+(1-\lambda _{1})a \bigr]< E(a,b)< H\bigl[\mu_{1}a+(1-\mu_{1})b, \mu_{1}b+(1-\mu_{1})a\bigr], \\ G\bigl[\lambda_{2}a+(1-\lambda_{2})b, \lambda_{2}b+(1- \lambda _{2})a\bigr]< E(a,b)< G\bigl[\mu_{2}a+(1- \mu_{2})b, \mu_{2}b+(1-\mu_{2})a\bigr] \end{gathered} $$

*hold for all* $a, b>0$ *with* $a\neq b$ *if and only if* $\lambda_{1}\leq 1/2-\sqrt{1-8/\pi^{2}}/2=0.2823\ldots$ , $\mu_{1}\geq1/2-\sqrt{2}/8=0.3232\ldots$ , $\lambda_{2}\leq1/2-\sqrt {1-64/\pi^{4}}/2=0.2071\ldots$ *and* $\mu_{2}\geq1/4$.

Let $p\in[1, \infty)$, $r\in(0, 1)$, $a=r$, $b=1-r^{2}={r^{\prime }}^{2}$, $\lambda=1/2-\sqrt{1-(2\sqrt{2}/\pi)^{4/p}}/2$ and $\mu=1/2-\sqrt{p}/(4p)$. Then (1.1) and Theorem 3.1 lead to Corollary 3.3 immediately.

### Corollary 3.3

*The double inequality*

$$ \begin{gathered} \frac{\sqrt{2}\pi}{4} \bigl(1+{r^{\prime}}^{2} \bigr)^{(1-p)/2} \biggl[4{r^{\prime}}^{2} + \biggl( \frac{8}{\pi^{2}} \biggr)^{2/p}r^{4} \biggr]^{p/4} \\ \quad< \mathcal{E}(r) < \frac{\sqrt{2}\pi}{4} \bigl(1+{r^{\prime }}^{2} \bigr)^{(1-p)/2} \biggl[\bigl(1+{r^{\prime}}^{2} \bigr)^{2}-\frac {r^{4}}{4p} \biggr]^{p/4} \end{gathered} $$

*holds for all* $r\in(0, 1)$ *and* $p\in[1, \infty)$.

## Results and discussion

In this paper, we provide the sharp bounds for the special quasi-arithmetic mean $E(a,b)$ in terms of the arithmetic mean $A(a,b)$ and geometric mean $G(a,b)$ with two parameters. As consequences, we present the best possible one-parameter harmonic and geometric means bounds for $E(a,b)$ and find new bounds for the complete elliptic integral of the second kind.

## Conclusion

In the article, we derive a new bivariate mean $E(a,b)$ from the quasi-arithmetic mean and provide its sharp upper and lower bounds in terms of the concave combination of arithmetic and geometric means.
